# Prognostic value of a novel myeloid-to-lymphoid ratio biomarker in advanced gastric cancer

**DOI:** 10.1007/s12094-024-03612-3

**Published:** 2024-08-14

**Authors:** Yuting Pan, Yue Ma, Guanghai Dai

**Affiliations:** 1 Medical School of Chinese PLA, Beijing, 100853 China; 2 Department of Medical Oncology, the First Medical Centre, Chinese PLA General Hospital, Beijing, 100853 China; 3 Department of Medical Oncology, the Fifth Medical Centre, Chinese PLA General Hospital, Beijing, 100853 China

**Keywords:** Gastric cancer, Immunotherapy, Myeloid to lymphoid lineage ratio, Prognostic biomarker

## Abstract

**Background:**

Currently, immune checkpoint inhibitors (ICIs) have excellent performance in the clinical treatment of advanced gastric cancer (AGC). However, precisely selecting AGC patients who can benefit from immunotherapy is an urgent difficulty. In this study, we investigated the immunoprognostic role of myeloid-to-lymphocyte ratio (M:L) in AGC patients.

**Methods:**

We collected information on 268 AGC patients who were hospitalized in the Department of Medical Oncology of PLA General Hospital from December 2014 to May 2021. The patients were divided into low M: L group (< 3.76) and high M:L group (≥ 3.76). Survival differences between different M: L level groups at baseline and after treatment were analyzed by methods such as Kaplan–Meier, Cox or Logistic regression model.

**Results:**

Progression free survival (PFS) (5.8 months vs. 3.4 months, *p* = 0.001) and overall survival (OS) (14.1 months vs. 9.0 months, *p* = 0.001) were significantly longer in the low M:L group than in the high M:L group. After analyses of Cox regression modeling it was concluded that M:L was an independent prognostic factor for PFS (HR 1.371 95%CI 1.057–1.777 *p* = 0.017) and OS (HR 1.352 95%CI 1.003–1.824 *p* = 0.048), respectively. Subsequent subgroup analyses performed across immunotherapy lines, regimens, PD-1 inhibitor agents, and age groups revealed a poorer prognosis in the high M:L group. Notably, an increase in the value of M:L after treatment significantly increased the risk of poor prognosis.

**Conclusions:**

M:L ≥ 3.76 is associated with poor prognostic outcomes in AGC patients receiving immunotherapy and may be a predictive biomarker of prognosis. This result needs to be confirmed by larger prospective studies.

**Supplementary Information:**

The online version contains supplementary material available at 10.1007/s12094-024-03612-3.

## Introduction

Gastric cancer (GC) ranks as the fifth most common cancer in the world with more than 1.08 million new cases per year. Meanwhile, GC is the fourth most common cause of tumor death. It is estimated that there are 768,793 deaths due to GC globally, with an overall global mortality rate of 7.7/100,000 [1]. The age-standardized incidence rate (ASIR) and age-standardized mortality rate (ASMR) of GC also vary significantly by region [2]. The hotspots for gastric cancer incidence and mortality lie in East Asia, Eastern Europe and South America [3].

The use of immune checkpoint inhibitors (ICIs) has been considered as a therapeutic option when patients with GC have undergone two or more lines of chemotherapy and have progressed again. There have been a number of clinical trials conducted on ICIs around the world, and the results, while affirming the efficacy of ICIs to a certain extent, have demonstrated that different populations have different sensitivities to the effects of ICIs [4–7]. Biomarkers that can predict the prognosis of immunotherapy are urgently sought to screen for patients who can truly benefit from it.

Currently, the biomarkers that are widely recognized by the medical community as being able to predict the prognosis of immunotherapy are PD-L1 CPS, microsatellite instability-high (MSI-H) and tumor mutation burden (TMB). Although these biomarkers can suggest better efficacy with ICIs to some extent, they inevitably have some limitations [8, 9]. To address the difficulties of low detection rates of the above biomarkers, researchers have shifted their focus to peripheral blood biomarkers that are easy to collect and simple to detect. For example, neutrophil-to-lymphocyte ratio (NLR), platelet-to-lymphocyte ratio (PLR), and prognostic nutritional index (PNI), to name a few, have been shown to be potentially effective surrogate indicators of efficacy of ICIs in tumor patients [10–12].

Among the various markers available in peripheral blood, the myeloid to lymphoid lineage ratio (M: L) is of interest because this combination of markers contains a more comprehensive picture of the tumor microenvironment (TME). The eosinophils and monocytes in this combination are rarely found in other peripheral blood tumor markers. Previously Soyano et al. did a study related to M: L prediction of prognosis in patients with advanced non-small cell lung cancer (NSCLC) [13]. Their results showed that patients with baseline M: L higher than 11.3 had significantly lower 12-month survival and were associated with an increased risk of death. And data from this study showed that M: L was significantly more associated with patient survival after immunotherapy than other common peripheral blood markers. Thus, the present study was a retrospective case analysis of 268 AGC patients who had received immunotherapy to investigate the role of M: L in predicting the immune prognosis of AGC patients and to provide a more specific and in-depth analysis in the context of real-world clinical conditions.

## Material and methods

### Research subjects

Clinical data, blood routine and blood biochemical results that were within seven days prior to receiving immunotherapy was collected from 268 patients who were diagnosed with AGC in the Chinese People’s Liberation Army General Hospital from January 2017 to April 2021 and analyzed retrospectively. Inclusion criteria: (1) The pathological diagnosis of the patient was definitively either GC grade III or IV; (2) Patients received multi-cycle immunotherapy (at least 2 treatment cycles); (3) During the treatment, the imaging efficacy evaluation of RECIST1.1 standard were completed multiple times (at least 1 evaluation completed); (4) Blood biochemical and routine results were completed within one week before the first use of ICIs. Exclusion criteria: (1) Patients with infection, autoimmune disease, or idiopathic thrombocytopenic purpura; (2) Patients that had no assessable lesions; (3) Patients with other complications such as other tumors, heart failure, liver and kidney dysfunction or other serious medical diseases; (4) Patients with missing blood routine and biochemical results within 1 week before immunotherapy. Patient-specific medication regimens and imaging evaluation times are available in Supplementary Material. All patients signed the informed consent form, and the study was approved by the independent ethics committee of the institution, and was conducted in accordance with the ethical principles of Helsinki Declaration according to local laws and regulatory requirements.

### Assessment

During immunotherapy, imaging examination was performed every 4–6 weeks, and the short-term curative effect was evaluated according to RECIST1.1 standard of solid tumor. The evaluation results of curative effect were progressive disease (PD), partial response (PR), complete remission (CR), and stable disease (SD) respectively. The short-term efficacy was evaluated by overall response rate (ORR) = (CR + PR)/total cases × 100%, and disease control rate (DCR) = (CR + PR + SD)/total cases × 100%. For long-term efficacy evaluation, progression free survival (PFS) was defined as the time from the first treatment to the confirmation of PD, death, or the last follow-up, and overall survival (OS) which was defined as the time from the start of immunotherapy to death.

### Pretreatment calculation of the M:L and the M:L cut-off value

We collected and recorded the absolute eosinophil count (AEC), absolute neutrophil count (ANC), absolute monocyte count (AMC) and absolute lymphocyte count (ALC) in peripheral blood samples at baseline (within the window period of 7 days before the first administration of immunotherapy) and calculated the M:L ratio. M:L was defined as the ratio of (ANC + AMC + AEC) to ALC. A receiver operating characteristic (ROC) curve is applied to elucidate the optimal cutoff value for M:L. We determined to take the 3.76 as the best cut-off value of M:L. Patients were then divided based on M:L into two groups: Low M:L group (< 3.76) and High M:L group (≥ 3.76).

### Statistical analysis

All data were processed by SPSS 26.0 and GraphPad Prism 9. Continuous variables were described using medians and ranges of maximum and minimum values. Categorical variables were described with the use of percentages. *χ*^2^ or Fisher’s exact test was carried out to evaluate the relationship between clinical response and M:L of AGC patients. The survival curve was depicted by Kaplan–Meier analysis. Logistic regression models and Cox proportional-hazard models were applied to assess the prognostic values of M:L for short-term curative effect and long-term efficacy, respectively. *P* values less than 0.05 (*P* < 0:05) were considered statistically significant.

## Results

### ROC analysis and grouping

M:L is graded based on a combination of ANC, AMC, AEC and ALC levels. In our study, the ROC curve was used to calculate the optimal cut-off value of M:L (3.76). ROC curve analysis was performed with death within 3 months of immunotherapy as the dependent variable. The results showed that the sensitivity of M:L was 0.794, the specificity was 0.598, and the AUC was 0.700 (*p* < 0.001)(Supplementary Fig. 1). AGC patients were divided into low M:L group (M:L < 3.76) and high M:L group (M:L ≥ 3.76) according to the optimal cut-off value of M:L.

### Baseline characteristics

**Fig. 1:**
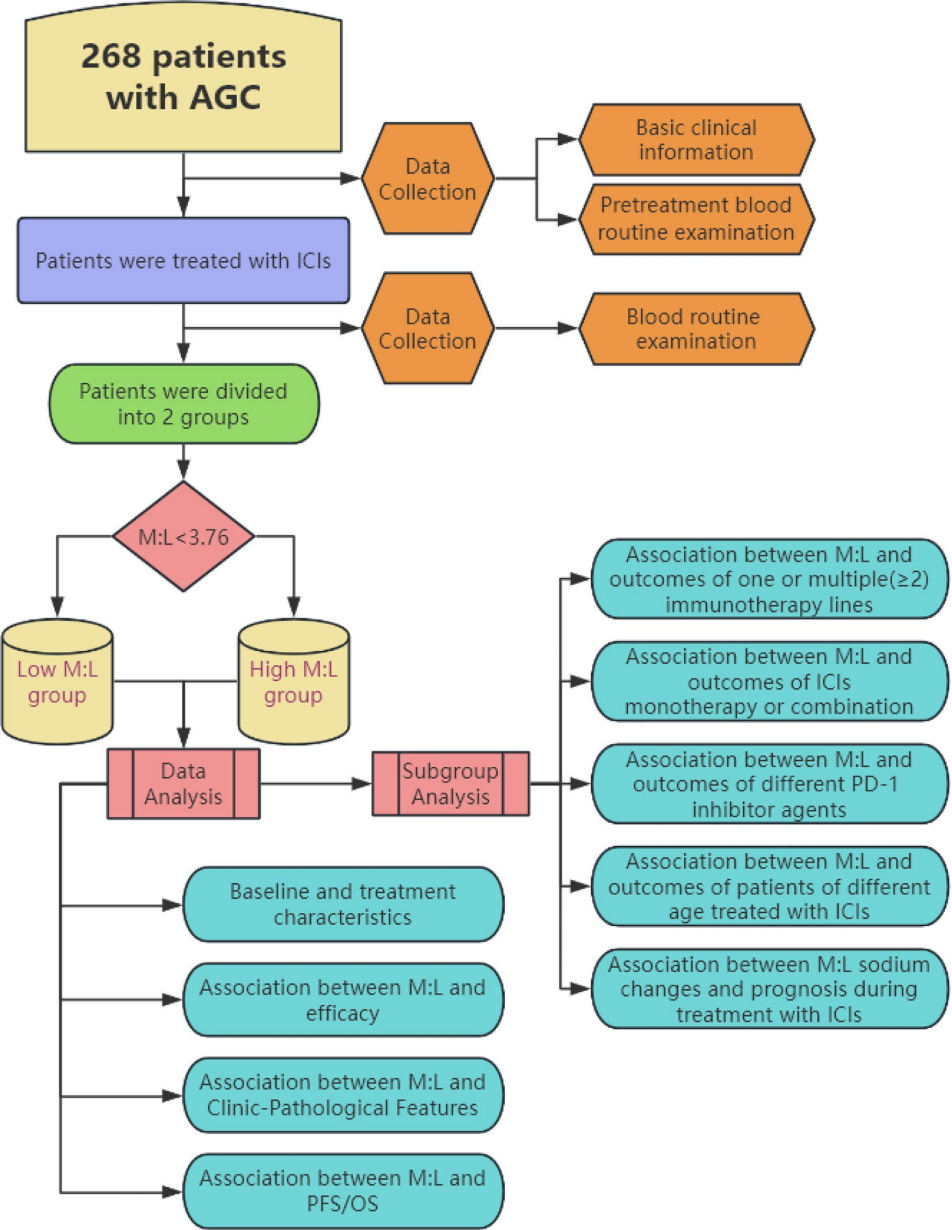
Study design flowchart. This retrospective case analysis study investigates the predictive role of M:L on immune prognosis in patients with advanced gastric cancer. Abbreviations: *AGC* advanced gastric cancer, *ICIs* immune checkpoint inhibitors, *M:L* myeloid to lymphoid lineage ratio, *PFS *progression-free survival, *OS *overall survival

A total of 268 patients were included according to inclusion and exclusion criteria. An overview of the study for all patients is shown in Fig. 1. All the baseline characteristics and treatment of patients with such conditions such as shown in Table 1. There were 147 AGC patients in the low M:L group, with a median age of 59 years, 110 males(74.8%), 37 females (25.2%), 57 patients(38.8%) with a history of smoking, 28 patients (19.0%) with more than 30 packs per year, and 2 patients (1.4%) Eastern Cooperative Oncology Group (ECOG) performance status (PS) was ≥ 2 points. Most had no pleural fluid(94.6%) or ascites(76.2%). No significant difference in tumor location in cardia, body/fundus and pylorus (27.9% vs 38.1% vs 32.7%) and the majority (57.1%) did not have liver metastases. Human epidermal growth factor receptor-2 (HER-2) expression was absent in more than half of the patients (66.7%). There were 121 patients in the high M:L group, with a median age of 61 years, 89 males(73.6%), 32 females(26.4%), 44 patients (36.4%) with a history of smoking, 20 patients (16.5%) with more than 30 packs per year, and 14 patients (11.6%) with ECOG PS ≥ 2 points. Most had no pleural fluid(90.9%) or ascites(74.4%) and HER-2 expression was absent (65.3%). No significant difference in tumor location in cardia, body/fundus and pylorus (26.5% vs 44.6% vs 28.9%) (Table 1).

**Table 1:** General data and clinical feature

Characteristics	Number of patients (%)			
	Overall (n = 268)	Low group (n = 147)	High group (n = 121)	*P*
Median age (range), years	59 (18-86)	59 (22-85)	61 (18-86)	
Sex				
Female	69 (25.7)	37 (25.2)	32 (26.4)	0.889
Male	199 (74.3)	110(74.8)	89 (73.6)	
Smoking history				
Yes	101 (37.7)	57 (38.8)	44 (36.4)	0.685
No	167 (62.3)	90 (61.2)	77 (63.6)	
Smoking exposure				
> 30 packs per year	48 (17.9)	28 (19.0)	20 (16.5)	0.593
≤ 30 packs per year	220 (82.1)	119 (81.0)	101 (83.5)	
ECOG PS				
≥ 2	16 (6)	2 (1.4)	14 (11.6)	＜0.001
0-1	252 (94)	145 (98.6)	107 (88.4)	
Pleural fluid				
Present	19 (7.1)	8 (5.4)	11 (9.1)	0.247
Absent	249 (92.9)	139 (94.6)	110 (90.9)	
Ascites				
Present	66 (24.6)	35 (23.8)	31 (25.6)	0.732
Absent	202 (75.4)	112 (76.2)	90 (74.4)	
Tumor_location				
Cardia	73 (27.2)	41 (27.9)	32 (26.5)	0.599
Body/Fundus	110 (41)	56 (38.1)	54 (44.6)	
Pylorus	83 (31)	48 (32.7)	35 (28.9)	
Unknown	2 (0.7)	2 (1.3)	0 (0)	
Liver metastasis				
No	149 (55.6)	84 (57.1)	65 (53.7)	0.575
Yes	119 (44.4)	63 (42.9)	56 (46.3)	
*HER-2*				
Present	34 (12.7)	19 (12.9)	15 (12.4)	0.956
Absent	177 (66)	98 (66.7)	79 (65.3)	
Unknown	57 (21.3)	30 (20.4)	27 (22.3)	
Lines of immunotherapy				
≥2	143 (53.4)	70 (47.6)	73 (60.3)	0.038
<2	125 (46.6)	77 (52.4 )	48 (39.7)	
PD-1 inhibition agents				
Pembrolizumab	36 (13.4)	17 (11.6)	19 (15.7)	0.048
Nivolumab	88 (32.8)	41 (27.9)	47 (38.8)	
Other	144 (53.7)	89 (60.5)	55 (45.5)	
ICIs combined with chemotherapy				
No	173 (64.6)	102 (69.4)	71 (58.7)	0.068
Yes	95 (35.4)	45 (30.6)	50 (41.3)	

*PD* progressive disease, *HER-2* human epidermalgrowth factor receptor-2, *PD-1* programmed cell death-1, *ECOG PS* eastern cooperative oncology group performance status scores, *ICIs* immune checkpoint inhibitors. The other PD-1 inhibitors are Sintilimab or Toripalimab

### Treatment characteristics

Among patients in the low M:L group, 17 patients (11.6%) were treated with pembrolizumab, 41 (27.9%) were treated with nivolumab, and the remaining 89 patients (60. 5%) were treated with other ICIs; 70 patients (47.6%) received multi-line immunotherapy; the remaining 77 patients (52.4%) received first-line immunotherapy; and 102 patients (69.4%) received monotherapy with ICIs only, while the remaining 45 patients (30.6%) received a combination of ICIs and chemotherapy. In the high M:L group, 19 patients (15.7%) were treated with pembrolizumab, 47 patients (38.8%) with nivolumab, and the remaining 55 (49.6%) were treated with other ICIs; 73 patients (60.3%) received multiple lines of immunotherapy; the remaining 48 patients (39.7%) received first-line immunotherapy; and 71 patients (58.7%) received monotherapy with ICIs only, while the remaining 50 patients (41.3%) received a combination of ICIs and chemotherapy (Table 1).

### Association between M:L and efficacy

**Fig. 2:**
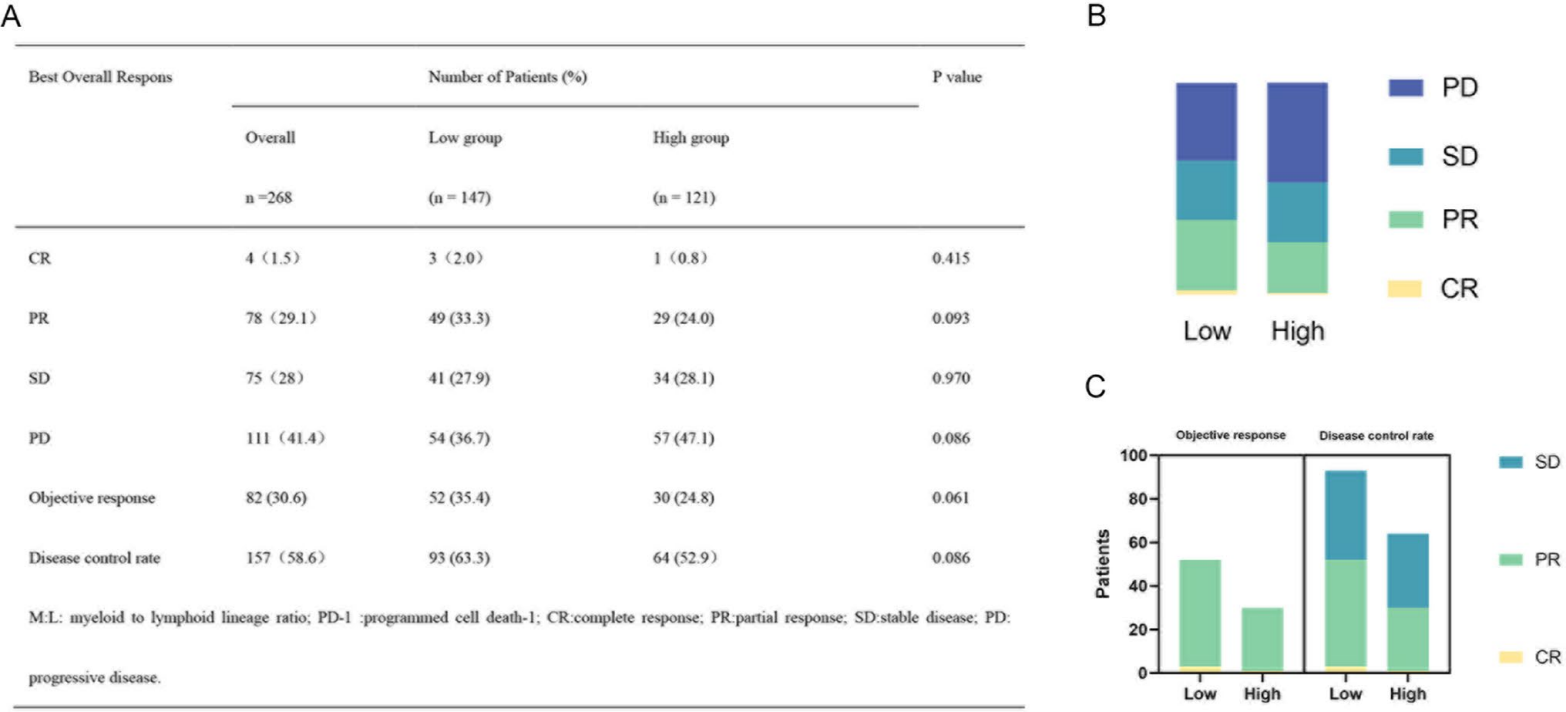
Relationship between low and high level groups of M:L and response to anti-PD-1 treatment. (A) Summary table of anti-PD-1 responses. (B) Proportion of anti-PD-1 responses. (C) Chart of anti-PD-1 response numbers. Abbreviations: M:L myeloid to lymphoid lineage ratio, PD-1 programmed cell death-1, CR complete response, *PR *partial response, *SD *stable disease, *PD *progressive disease

According to the evaluation standards of RECIST 1.1, the best response (BOR) of the low M:L group, were as follows: PD accounted for 36.7% (54 patients) vs CR 2.0% (3 patients), PR 33.3% (49 patients), and SD 27.9% (41 patients). The study evaluated the optimal efficacy of the high M:L group, the results of which were as follows: PD accounted for 47.1% (57 patients) vs CR 0.8% (1 patient), PR 24.0% (29 patients), and SD 28.1% (34 patients). The low and high M:L groups did not show a clear difference in DCR ( 63.3% vs 52.9%, *p* = 0.086), or in ORR (35.4% vs 24.8%; *p* = 0.061) (Fig. 2).

### Association between M:L and clinic-pathological features

**Fig. 3:**
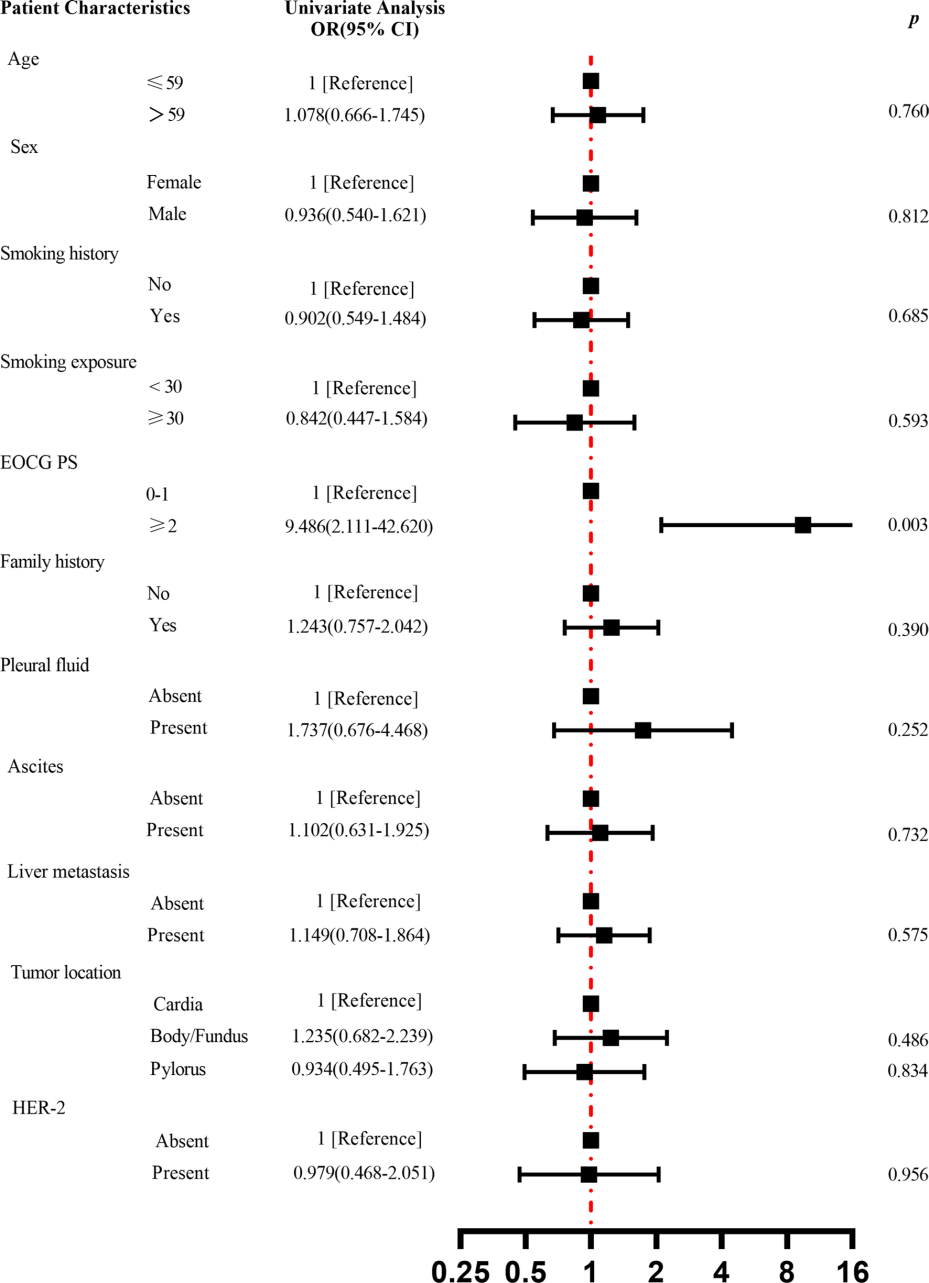
Clinical–pathological features of the patients according to the M:L. The forest plot demonstrates the results of the logistic regression model analysis of clinical-pathological features versus M:L. The EOCG PS ≥ 2 increased the risk of high M:L with statistical significance (OR 9.486; 95% CI 2.111–42.620; *p* = 0.003). Abbreviations: *M:L* myeloid to lymphoid lineage ratio, *ECOG PS* Eastern Cooperative Oncology Group performance status score, *HER-2 *human epidermal growth factor receptor-2

We used logistic regression equations to analyze the correlation between basic characteristics such as age, sex, smoking history, smoking exposure, ECOG PS, family history, pleural fluid, ascites, liver metastases, tumor location, and HER-2 expression with M:L, and the results are shown in Fig. 3. The EOCG PS ≥ 2 increased the risk of high M:L with statistical significance (OR 9.486; 95% CI 2.111–42.620; *p* = 0.003). However, there was no correlation between other clinical characteristics of the patients (e.g. age, gender, etc.) and high M:L.

### Association between M:L and PFS

**Fig. 4:**
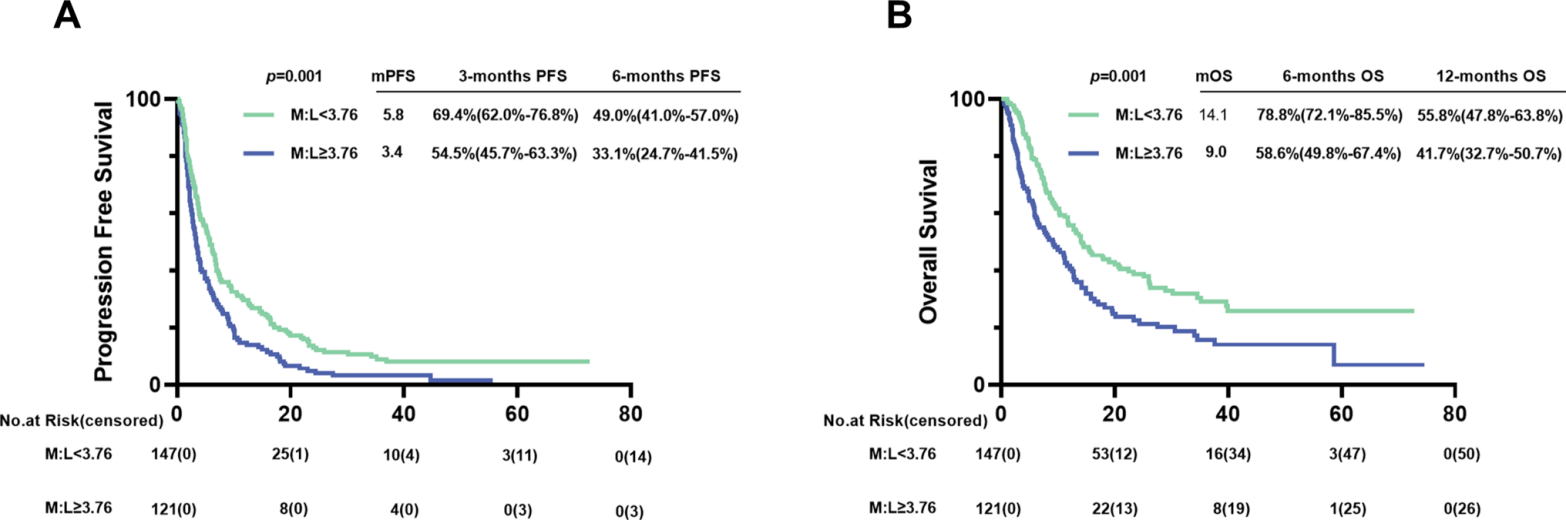
PFS **A** and **B** OS of AGC patients treated with PD-1 inhibitor. Kaplan–Meier analysis showed that the low M:L group was associated with a better prognosis. Abbreviations: *M:L* myeloid to lymphoid lineage ratio, *PFS *progression free survival, OS overall survival, AGC advanced gastric cancer

**Fig. 5:**
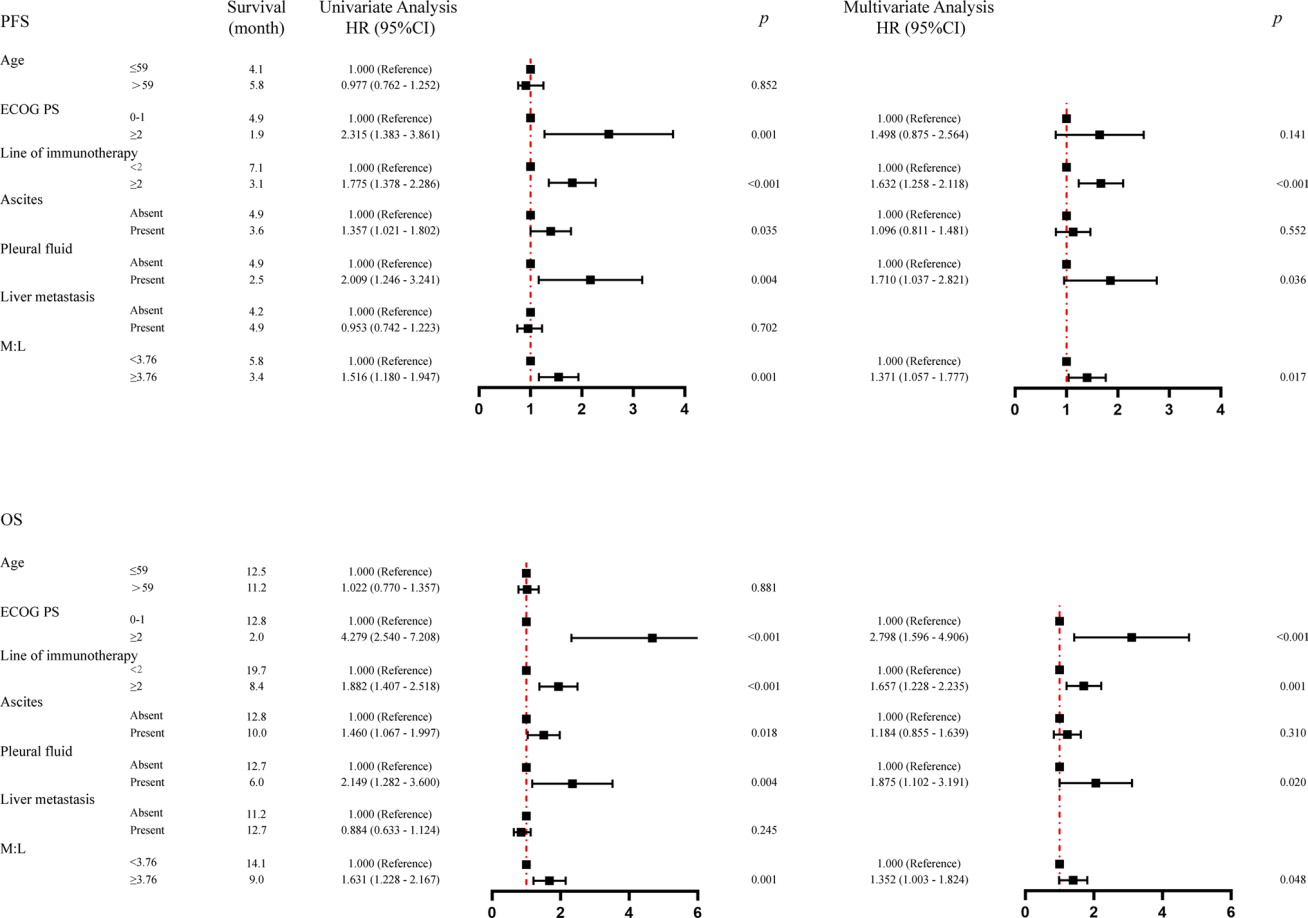
Univariate and multivariate analyses of factors associated with overall survival and progression-free survival. Multivariate COX correction models showed that high M:L was associated with shorter PFS and OS. Abbreviations: *PFS* progression-free survival, OS overall survival *ECOG PS *Eastern Cooperative Oncology Group performance status scores, *M:L* myeloid to lymphoid lineage ratio

As of July 1st, 2023, 17 AGC patients did not progress after immunotherapy. The median PFS of the low M:L group was 5.8 months, and the high M:L group was 3.4 months. Survival analysis showed that AGC patients in the low M:L group had longer PFS benefit advantages than those in the high M:L group (*p* = 0.001) (Fig. 4A). Cox univariate and multivariate analyses were performed with disease progression in AGC patients after immunotherapy as the dependent variable, and M:L, ECOG PS, lines of immunotherapy, and ascites and pleural fluid status as the independent variables, and the results are shown in Fig. 5. The high M:L group (HR = 1.371; 95% CI, 1.057–1.777; *p* = 0.017) was independently associated with an increased risk of progression after cox multivariate model adjustment.

### Association between M:L and OS

As of July 1st, 2023, 76 AGC patients were still alive. The median OS of the low M:L group was 14.1 months, and the high M:L group was 9.0 months. Survival analysis showed that AGC patients the low M:L group had longer OS benefit advantages than those in the high M:L group (*p* = 0.001) (Fig. 4B). Cox univariate and multivariate analyses were performed with death in AGC patients after immunotherapy as the dependent variable, and M:L, ECOG PS, lines of immunotherapy, and ascites and pleural fluid status as the independent variables, and the results are shown in Fig. 5. The high M:L group (HR = 1.352; 95% CI, 1.003–1.824; *p* = 0.048) was independently associated with an increased risk of death after cox multivariate model adjustment.

### Association between M:L and outcomes of one or multiple (≥ 2) immunotherapy lines: subgroup analysis

Significant improvements in PFS and OS were observed in patients treated with first-line ICIs after adjustment for cox multivariate modeling. Therefore, we conducted subgroup analysis according to the number of lines of immunotherapy. Of the 125 patients who received first-line immunotherapy, 77 (61.6%) were in the low M:L group and 48 (38.4%) were in the high M:L group. Patients in the low M:L group had significantly improved PFS and OS compared with those in the high M:L group(7.3 vs 4.9 months, 23.3 vs 15.8 months;* p* = 0.038, *p* = 0.124), but the OS results were not statistically significant(Supplementary Fig. 2A, B). Of the 143 AGC patients who received subsequent lines of ICIs, 70 (49.0%) patients were in the low M:L group and 73 (51.0%) were in the high M:L group. Patients in the low M:L group had a statistically significant prolongation of PFS and OS compared to those in the high M:L group(3.6 vs 2.8 months, 10.2 vs 6.4 months;* p* = 0.046, *p* = 0.011) (Supplementary Fig. 2C, D).

### Association between M:L and outcomes of ICIs monotherapy or ICIs treatment combined with chemotherapy: subgroup analysis

Based on the real-world clinical regimens, we categorized immunotherapy regimens into PD-1 inhibitor monotherapy regimens and PD-1 inhibitor combined with chemotherapy regimens, and performed subgroup analyses based on the data obtained. Of the 173 AGC patients treated with PD-1 inhibitor monotherapy, 102 (59.0%) patients were in the low M:L group and 71 (41.0%) patients were in the high M:L group. Patients in the low M:L group had significant PFS (6.4 vs 3.6 months, *p* = 0.002) and OS (16.1 vs 11.0 months, *p* = 0.013) benefits compared to patients in the high M:L group (Supplementary Fig. 3A, B). Among 95 AGC patients treated with PD-1 inhibitor in combination with chemotherapy, 45 (47.4%) patients belonged to the low M:L group and 50 (52.6%) patients belonged to the high M:L group. Compared to patients in the high M:L group, patients in the low M:L group had a statistically significant prolongation of OS (9.7 vs 8.2 months, *p* = 0.071), but there was no statistically significant difference in the prolongation of PFS (3.3 vs 2.9 months, *p* = 0.289)( Supplementary Fig. 3C, D).

### Associations between M:L and outcomes of patients treated with different PD-1 inhibitor agents: subgroup analysis

In order to more closely match the reality of clinical drug use, we further analyzed the specific differences between PD-1 inhibitor agents in depth. In this study, PD-1 inhibitor agents were categorized into three groups: pembrolizumab, nivolumab, and others. Of the 36 individuals on pembrolizumab, 17 (47.2%) were in the low M:L group and 19 (52.8%) were in the high M:L group. Patients in the low M:L group had longer FPS (7.4 months vs. 6.6 months, *p* = 0.089) and OS (16.1 months vs. 11.6 months, *p* = 0.109), but were not statistically significant(Supplementary Fig. 4A, B). Of the 88 individuals on nivolumab, 41 (46.6%) were in the low M:L group and 47 (53.4%) in the high M:L group. Patients in the low M:L group had significantly longer PFS (4.0 months vs. 2.2 months, *p* = 0.023) and OS (7.7 months vs. 4.8 months, *p* = 0.01) than those in the high M:L group(Supplementary Fig. 4C, D). The clinical prognostic benefit was not statistically significant in patients in the low M:L group among patients using other PD-1 inhibitor agents(Supplementary Fig. 4E, F).

### Associations between M:L and outcomes of patients of different ages treated with ICIs: subgroup analysis

As society gradually enters the ageing era, the situation of the elderly is receiving more and more attention. In this study, we used 59 years of age as the cut-off value (median age of the patients), and divided the age group of 59 years and above into the elderly group, and the age group of less than 59 years into the middle-aged and young group for subgroup analysis. Of the 139 patients in the elderly group, 75 patients (54.0%) belonged to the low M:L group and 64 (46.0%) to the high M:L group. There was a statistically significant benefit in PFS (6.7 vs 3.3 months, *p* = 0.047) but no statistically significant difference in OS prolongation (13.1 vs 9.4 months, *p* = 0.225) in patients in the low M:L group compared to those in the high M:L group(Supplementary Fig. 5A, B). Of the 129 patients in the young and middle-aged group, 72 patients (55.8%) belonged to the low M:L group and 57 (44.2%) to the high M:L group. Patients in the low M:L group showed statistically significant benefit in both PFS (4.6 vs 3.6 months, *p* = 0.013) and OS (15.5 vs 9.0 months, *p* < 0.001) compared to those in the high M:L group(Supplementary Fig. 5C, D).

### Relationship between M:L changes during immunotherapy and prognosis: subgroup analysis

After evaluating the relationship between changes in M:L and immune prognosis in AGC patients at different times after receiving immunotherapy, we found that M:L data were available for 225 patients 4 weeks after the first immunotherapy, of which 113 patients (50.2%) had an increase in M:L values compared with baseline(δ 4 week > 0), and 112 patients (49.8%) did not have an increase or decrease in M:L values compared with baseline(δ 4 week ≤ 0). In contrast, 8 weeks after the first immunotherapy, data were available for 207 patients, of whom 106 (51.2%) had increased M:L values from baseline(δ 8 week > 0) and 101 (48.8%) had the same or decreased M:L values from baseline(δ 8 week ≤ 0)( Supplementary Fig. 6). We found that An increase in patients’ M:L values at 4 weeks of initial immunotherapy was statistically significantly associated with the occurrence of disease progression after 3 months(OR 1.091, 95%CI 1.028–1.157, *p* = 0.004)(Supplementary Table 1). Increased M:L values in patients at 8 weeks of initial immunotherapy were statistically significantly associated with the occurrence of death after 6 months (OR 1.089, 95%CI 1.011–1.173, *p* = 0.024) (Supplementary Table 2).

## Discussion

Peripheral blood is a non-invasive source for exploring potential biomarkers for ICIs. Although associations of peripheral blood biomarkers with clinical benefit and survival have been observed, the mechanisms behind them have not been clarified. One study of classical Hodgkin’s lymphoma analyzed circulating lymphocytes and plasma proteins in relation to clinical parameters and treatment efficacy, suggesting that immune modulation of lymphocytes in the TME may influence biomarkers in peripheral blood [14]. Peripheral blood ALC and AMC counts have been used as biomarkers of the tumor microenvironment and immunosurveillance in a variety of myeloid and lymphoid hematological malignancies [15–17]. These studies have demonstrated that peripheral blood biomarkers can predict immune prognosis by responding to the immune status of the tumor microenvironment.

There are numerous types of cells in the tumor microenvironment, including immune cells recruited from the bone marrow and expanded from some of the resident cells at the site of origin, as well as other stromal cells of controversial origin, such as fibroblasts and neural and vascular endothelial cells. It is these components of the microenvironment that not only facilitate tumor growth and expansion, but also help to free tumor cells from host immune surveillance and increase their resistance to cancer treatment [18]. The immune-related cells in the tumor microenvironment of gastric cancer are tumor-associated macrophages (TAM), myeloid-derived suppressor cells (MDSCs), and tumor-infiltrating lymphocytes (TILs). Among them, MDSCs are regulatory immune cells associated with chronic inflammation and tumor sites, and inhibit CD8 + T-cell function through the expression of PD-1 and CTLA-4. ICIs can block the inhibitory effect of MDSCs on CD8 + T-cells, which leads to anti-tumor effects [19]. TILs consist of T cells, B cells, and natural killer (NK) cells, while T cell subsets include CD8 + cytotoxic T cells, CD4 + T helper cells, FOXP3 + regulatory T cells, memory T cells, and NK cells.TILs infiltrate stroma and tumor cells, modulate host immune responses to tumor cells, and are significant in combating tumors. However, in some tumor microenvironments, the anti-tumor immune response is inhibited by upregulation of PD-L1 or CTLA-4 expression.

As easily accessible and non-invasive tests, studies correlating peripheral blood biomarkers with clinical benefit have been reported. For example, baseline values such as low absolute low absolute neutrophil count, low NLR, low absolute monocyte count, low myeloid suppressor cell frequency, high FoxP3 + Treg cell frequency, high lymphocyte frequency, and high eosinophil count have been associated with improvements in OS and PFS in ipilimumab-treated melanoma patients. The NLR has also been associated with improved OS and PFS in clinical prognosis in melanoma patients treated with pembrolizumab and NSCLC patients treated with nivolumab. Others, such as PLR, derived neutrophil-to-lymphocyte ratio (dNLR), and lung immune prognostic index (LIPI), have been proven in clinical trials as peripheral blood biomarkers to predict prognosis. In contrast, one of the advantages of the M:L scoring index involved in this study is that it contains a more comprehensive set of relevant indicators of response to TME, including eosinophils and monocytes, which are not found in other peripheral blood biomarkers.

Eosinophils have been reported to serve as predictive markers for immunotherapy in clinical studies of a variety of solid tumors, such as gastric cancer, colorectal cancer, lung cancer, breast cancer, and malignant melanoma, among others [20–24]. Activated tumor-infiltrating eosinophils produce high levels of chemokines, such as CCL5, CXCL9, and CXCL10, which attract co-metastatic CD8 + T cells to the tumor, leading to tumor rejection and prolonged survival [25]. Through the production of cytokines such as transforming growth factor *β*, C–C motif chemokine ligand 2, and C–C motif chemokine ligand 17, neutrophils are able to initiate and participate in complex crosstalk with other immune cells, leading to an immunosuppressive environment [26]. As myeloid precursor cells, monocytes can replenish the number of TAMs and monocytic MDSCs (M-MDSCs), as well as other myeloid cells, to support suppressive TME production and improve T-cell responses. Inhibition of retinoic acid production or signaling increases monocyte-derived dendritic cells (DCs), enhances T-cell-dependent antitumor immunity, and acts synergistically with ICIs [27]. *γ*-Aminobutyric acid (GABA) promotes the differentiation of monocytes into anti-inflammatory macrophages, which secrete IL-10 and suppress CD8 + T-cell function [28]. ICIs (PD-1, TIM-3, CTLA-4) involved in inhibiting T-cell activation/proliferation of TIL-expressing solid tumors may lead to peripheral lymphopenia. Tremelimumab, on the other hand, rapidly restores effector and memory CD4 + and CD8 + T-cell pools and T-cell receptor (TCR)-dependent T-cell proliferation in patients with advanced melanoma combined with severe lymphopenia [29, 30].

Therefore, for the first time, we selected the optimal critical value of M:L of 3.76 by plotting the ROC curve using the complex M:L indexes of AEC, ANC, AMC, and ALC as a predictor, and the "death" of AGC patients after 3 months of immunotherapy as an outcome variable. AGC patients with M:L values less than 3.76 were defined as the low M:L group; those above 3.76 were defined as the high M:L group. The results concluded that AGC patients in the baseline low M:L group had a significant improvement in both PFS and OS, which was statistically associated with an immune prognostic benefit. To the best of our knowledge, this is the first study to report that peripheral blood M:L is associated with immune prognosis in AGC patients. In the study by Soyano et al. 12-month OS was significantly lower in patients with increased baseline M:L compared to those with low baseline M:L (22.4% vs 47.0%; *p* < 0.001), but the decrease in PFS was not statistically significant (*p* = 0.11) [13]. Our study data showed statistically significant prolongation of both OS (14.1 months vs 9.0 months; *p* = 0.001) and PFS (5.8 months vs 3.4 months; *p* = 0.001) in patients with increased baseline M:L. In the study by Soyano et al., multifactorial Cox regression modeling analysis showed that patients with increased baseline M:L were associated with an increased risk of death (HR 1.04; 95%CI 1.02–1.06; *p* < 0.001), but the association with an increased risk of disease progression was not statistically significant (HR 1.36; 95%CI 0.91 −2.03; *p* = 0.13) [13]. Data from the present study showed that patients with increased baseline M:L were statistically significantly associated with both increased risk of death (HR 1.37; 95%CI 1.06–1.78; *p* = 0.017) and increased risk of disease progression (HR 1.35; 95%CI 1.01–1.82; *p* = 0.048). In the study by Soyano et al. 4 weeks after the first immunotherapy treatment, M:L change values were not statistically associated with disease progression after 3 months (OR 1.02; 95%CI 0.96–1.09; *p* = 0.52) [13]. However, a positive conclusion was drawn in our study (OR 1.09; 95%CI 1.03–1.16; *p* = 0.004). And we further concluded that the value of change in M:L after 8 weeks of the first immunotherapy was statistically associated with increased risk of death after 6 months (OR 1.09; 95%CI 1.01–1.17; *p* = 0.024). The reason for the difference between the results of the two studies may be the heterogeneity between AGC and NSCLC, and the patients’ status of immune response. This limits the application of the M:L index to AGC patients for the time being, pending larger prospective studies to explore its application in other stages of gastric cancer or even other tumor types. In addition, we found that immunotherapy with ICIs at multiple treatment lines significantly increased the risk of poor clinical prognosis, as did the risk of poor clinical prognosis in patients with pleural fluid status. Subsequently, we performed subgroup analyses and found that patients in the high M:L group did not achieve any real clinical survival benefit (statistically significant shortening of at least one of the OS or PFS) across different immunotherapy lines, regimens, PD-1 inhibitor agents, and age groups.

There are some limitations in this study: (1) The current study is a single-center, small-sample retrospective study with low level of evidence and possible bias. Therefore, these preliminary results need to be further verified by prospective research; (2) Lack of control group of patients who did not receive PD-1 inhibitor treatment; (3) Due to the large amount of missing data, the correlation between peripheral blood complex index combined with the expression of PD-L1 and the prognosis of immunotherapy was not discussed; (4) The number of lines of immunotherapy and the specific regimen of the eligible patients varied; (5) The results of the ML indicator were affected by a variety of factors in clinical practice, and the cut-off values of the indicators were calculated based on previous reports; (6) Data from public domain databases or other institutions were not used for external validation to enhance the robustness of the data; (7) This study was confined to the analysis of peripheral blood samples, and further assessment of clinical outcomes of patients with tumors should also be integrated with genomic and imaging tests.

## Conclusion

Our study shows for the first time that baseline M:L is an independent predictor of immunotherapy prognosis in AGC patients. The composite biomarker of M:L can screen out patients with potentially better prognosis after PD-1 treatment. This inflammatory indicator can help clinicians identify AGC patients who are more likely to benefit from anti-PD-1 treatment before treatment begins. In addition, as the evaluation of routine properties, the marker can be more easily applied to clinical practice.

## Supplementary Information

Below is the link to the electronic supplementary material.

Supplementary file1 (DOCX 1237 KB)

## Data Availability

The data presented in this study are available on request from the corresponding author. The data are not publicly available due to future planned analysis.

## Abbreviations

GC: Gastric cancer
ASIR: Age-standardized incidence rate
ASMR: Age-standardized mortality rate
ICIs: Immune checkpoint inhibitors
AGC: Advanced GC
PD-L1: Programmed cell death 1 ligand 1
CPS: Combined positive score
MSI-H: Microsatellite instability-high
TMB: Tumor mutation burden
NLR: Neutrophil-to-lymphocyte ratio
PLR: Platelet-to-lymphocyte ratio
PNI: Prognostic nutritional index
HSPCs: Hematopoietic stem and progenitor cells
ML: Myeloid to lymphoid lineage ratio
TME: Tumor microenvironment
NSCLC: Non-small cell lung cancer
PD: Progressive disease
PR: Partial response
CR: Complete remission
SD: Stable disease
ORR: Overall response rate
DCR: Disease control rate
PFS: Progression free survival
OS: Overall survival
AEC: Absolute eosinophil count
ANC: Absolute neutrophil count
AMC: Absolute monocyte count
ALC: Absolute lymphocyte count
ROC: Receiver operating characteristic
ECOG: Eastern Cooperative Oncology Group
PS: Performance Status
HER-2: Human epidermal growth factor receptor-2
BOR: Best response
TAM: Tumor-associated macrophages
MDSCs: Myeloid-derived suppressor cells.
TILs: Tumor-infiltrating lymphocytes
NK: Natural killer
dNLR: Derived neutrophil-to-lymphocyte ratio
LIPI: Lung immune prognostic index
M-MDSCs: Monocytic MDSCs
DCs: Dendritic cells
GABA: γ-Aminobutyric acid
TCR: T-cell receptor
MLR: Monocyte-to-lymphocyte ratio
EFS: Event-free survival

## Declarations

### Registry and the registration no. of the study/trial

N/A.

### Animal studies

N/A.
