# Supplementary material for: Prognostic value of a novel myeloid-to-lymphoid ratio biomarker in advanced gastric cancer
Source: Clin Transl Oncol. 2024 Aug 14;27(3):1118–30. doi: 10.1007/s12094-024-03612-3 (PMC11914242; doi:10.1007/s12094-024-03612-3)
Supplement: Supplementary file 1 — Supplementary file1 (DOCX 1237 KB) [file 12094_2024_3612_MOESM1_ESM.docx]

**Supplemental data**

**Prognostic Value of a Novel Myeloid-to-Lymphoid Ratio Biomarker in Advanced Gastric Cancer**

Yuting Pan, Yue Ma, Guanghai Dai

**Contents**

Treatment Regimens.. 3

Imaging Efficacy Evaluation..... 3

Supplementary Figure 1..... 4

Supplementary Figure 2..... 5

Supplementary Figure 3..... 6

Supplementary Figure 4..... 7

Supplementary Figure 5..... 8

Supplementary Figure 6..... 9

Supplementary Table 1..... 10

Supplementary Table 2.... 10

# Treatment Regimens

The types and doses of ICIs were as follows: (1) Sintilimab was injected intravenously 200 mg once every 3 weeks. (2) Toripalimab was injected intravenously 240 mg once every 3 weeks. (3) The recommended dose of pembrolizumab injection for intravenous infusion was a dose of 3 mg/kg, administered once every 3 weeks. (4) The recommended dose of nivolumab injection for intravenous infusion was a dose of 2 mg/kg, administered once every 2 weeks.

The chemotherapy regimens include (1) XELOX regimen: capecitabine (1,000 mg/m²) was used 2 times a day orally after breakfast and dinner for 14 consecutive days with 7 days of rest as a treatment cycle. Oxaliplatin (130 mg/m²) was added on the first day of each cycle by intravenous injection. (2) SOX regimen: tiggio (40–60 mg) was used 2 times a day orally after breakfast and dinner for 14 consecutive days with 7 days of rest as a treatment cycle. Oxaliplatin (130 mg/m²) was added on the first day of each cycle by intravenous injection. (3) DCF regimen: docetaxel (75mg/m²), cisplatin (75 mg/m²), and fluorouracil (750 mg/m²) were applied by intravenous injection. On the first day of every course, each course lasted 21 days. (4) The combined regimen of irinotecan and oxaliplatin: irinotecan (180 mg/m²) and oxaliplatin (130 mg/m²) were applied by intravenous injection. On the first day of every course, each course lasted 14 days. (5) The combined regimen of irinotecan and raltitrexed: irinotecan (180 mg/m²) and raltitrexed (3 mg/m²) were applied by intravenous injection. On the first day of every course, each course lasted 14 days. (6) Others. The choice of the above regimens was based on the patient’s pathological stage and general health conditions.

# Imaging Efficacy Evaluation

The first imaging evaluation of nivolumab was carried out 2–4 weeks after the 3rd intravenous injection; nevertheless, the evaluation of toripalimab, sintilimab, and pembrolizumab was carried out 3–5 weeks after the 2nd intravenous injection.

**
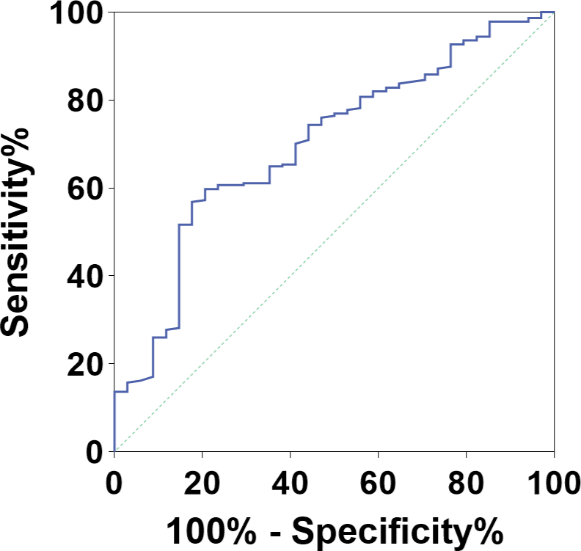
**

**Supplementary Figure 1.** ROC curves for pretreatment M:L to assess 3-month death rates. ROC curve was used to calculate the optimal cut-off value of M:L (3.76). ROC curve analysis was performed with death within 3 months of immunotherapy as the dependent variable. The results showed that the sensitivity of M:L was 0.794, the specificity was 0.598, and the AUC was 0.700 (p<0.001). Abbreviations: ROC: receiver operator characteristic; M:L: myeloid to lymphoid lineage ratio; blue line: M:L; green line: reference line.


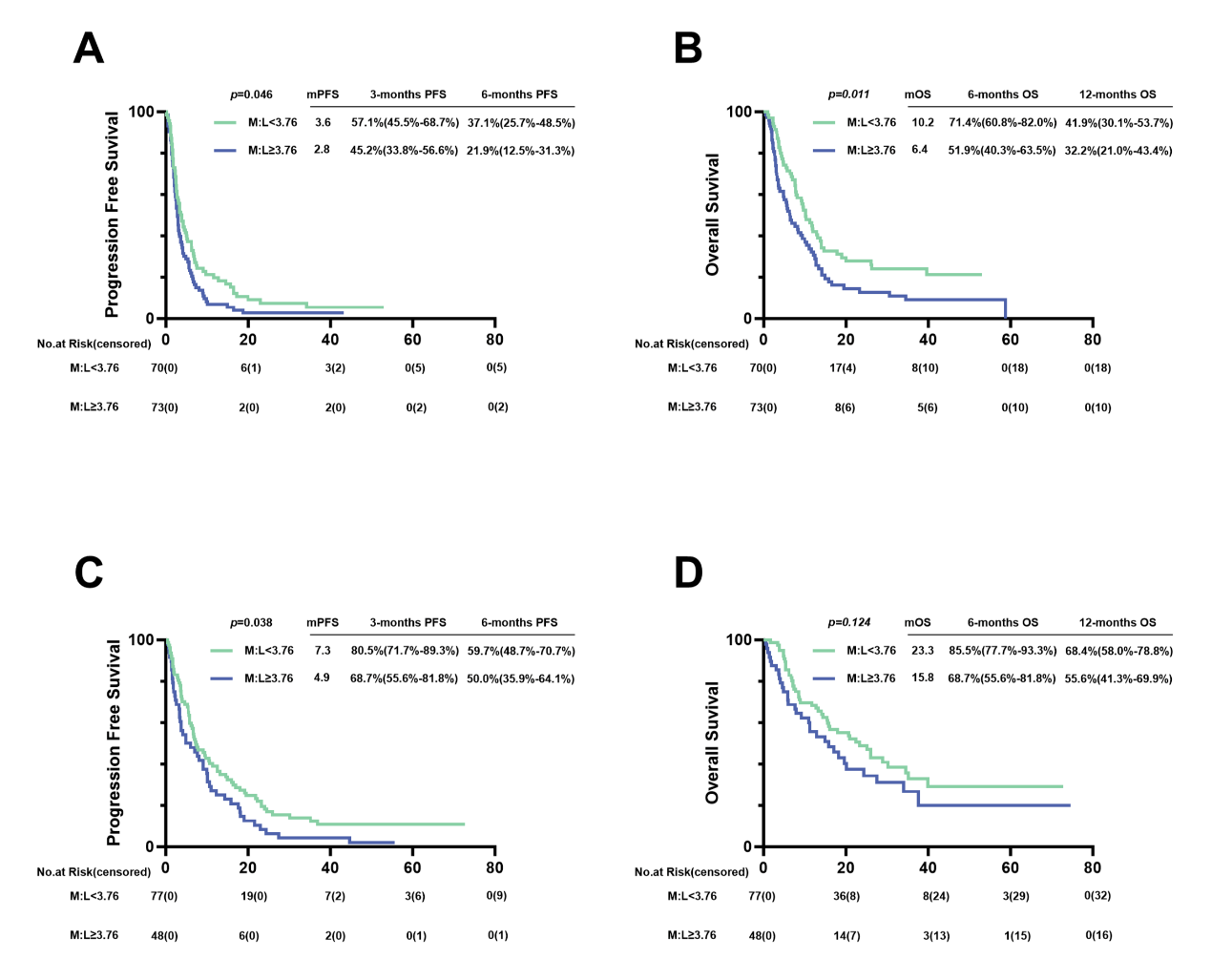


**Supplementary Figure 2.** PFS (A) and OS (B) of the 1st line of patients with AGC, receiving PD-1 inhibitor cohort. PFS (C) and OS (D) of the multi-line of patients with AGC, receiving PD-1 inhibitor cohort. Subgroup analysis of different immunotherapy lines showed that high M:L was associated with poor clinical prognosis. Abbreviations: PFS: progression free survival; OS: overall survival; PD-1: programmed cell death-1.


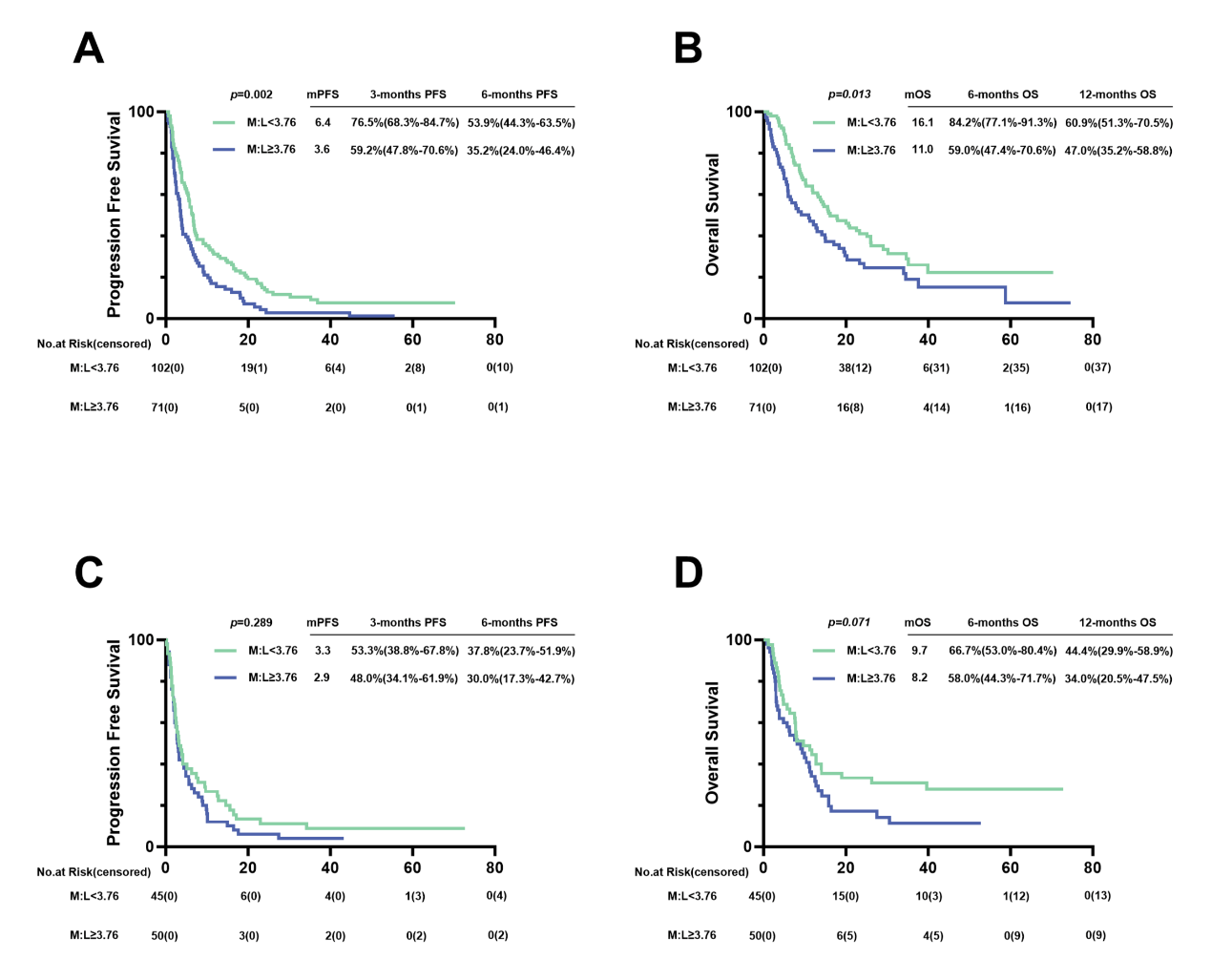


**Supplementary Figure 3.** PFS (A) and (B) OS of AGC patients treated with PD-1 inhibitor monotherapy. PFS (C) and (D) OS of AGC patients treated with PD-1 inhibitor combined with chemotherapy. Subgroup analysis of different immunotherapy regimens showed that high M:L was statistically significantly associated with poor clinical prognosis in patients treated with PD-1 inhibitor monotherapy. Abbreviations: PFS: progression free survival; OS: overall survival; PD-1: programmed cell death-1.


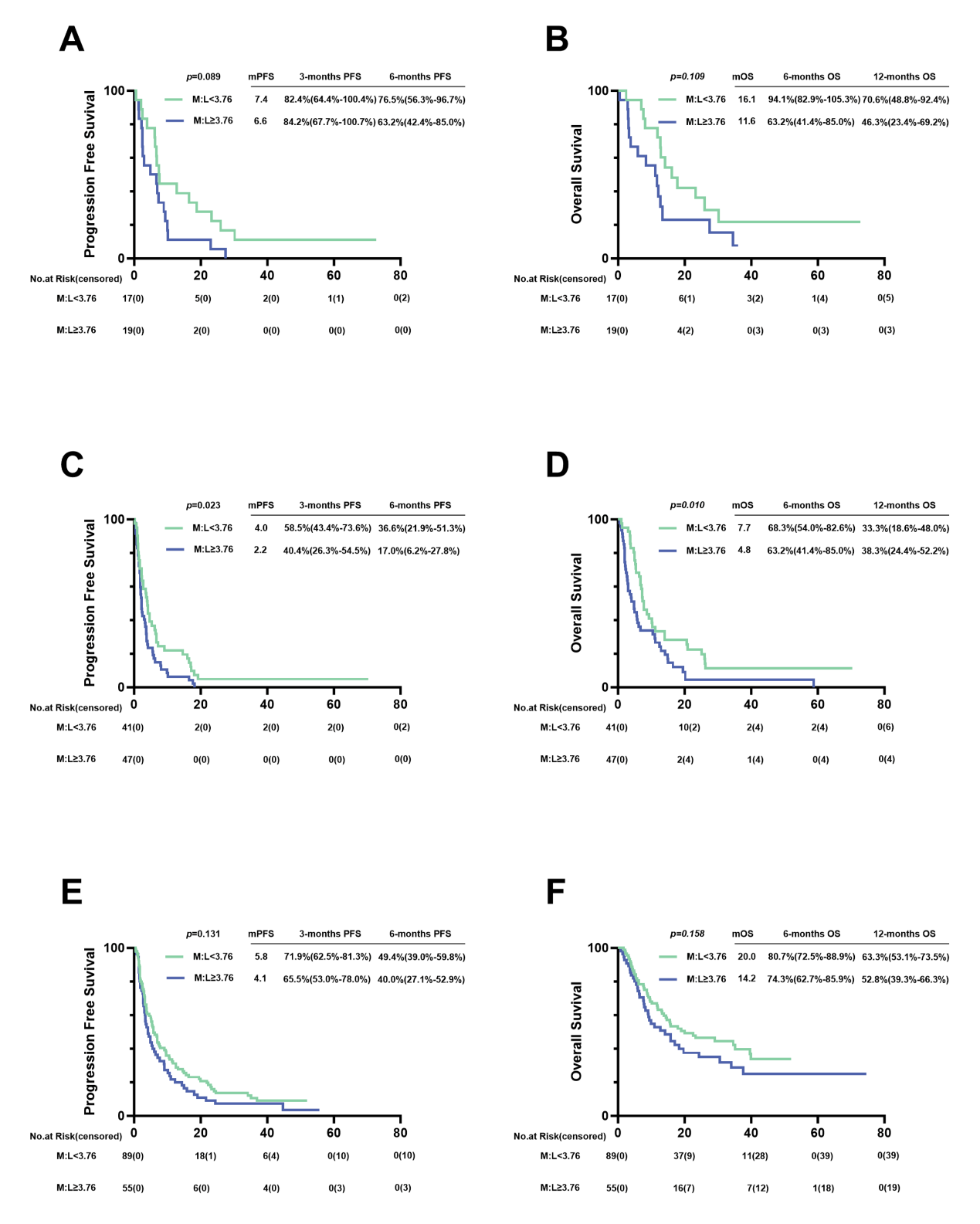


**Supplementary Figure 4.** PFS (A)and (B) OS of AGC patients treated with pembrolizumab. PFS (C)and (D) OS of AGC patients treated with nivolumab. PFS (E)and (F) OS of AGC patients treated with other PD-1 inhibitor agents. Subgroup analysis of different immunotherapy agents showed that high M:L was statistically significantly associated with poor clinical prognosis in patients immunized with nivolumab. Abbreviations: PFS: progression free survival; OS: overall survival; PD-1: programmed cell death-1.


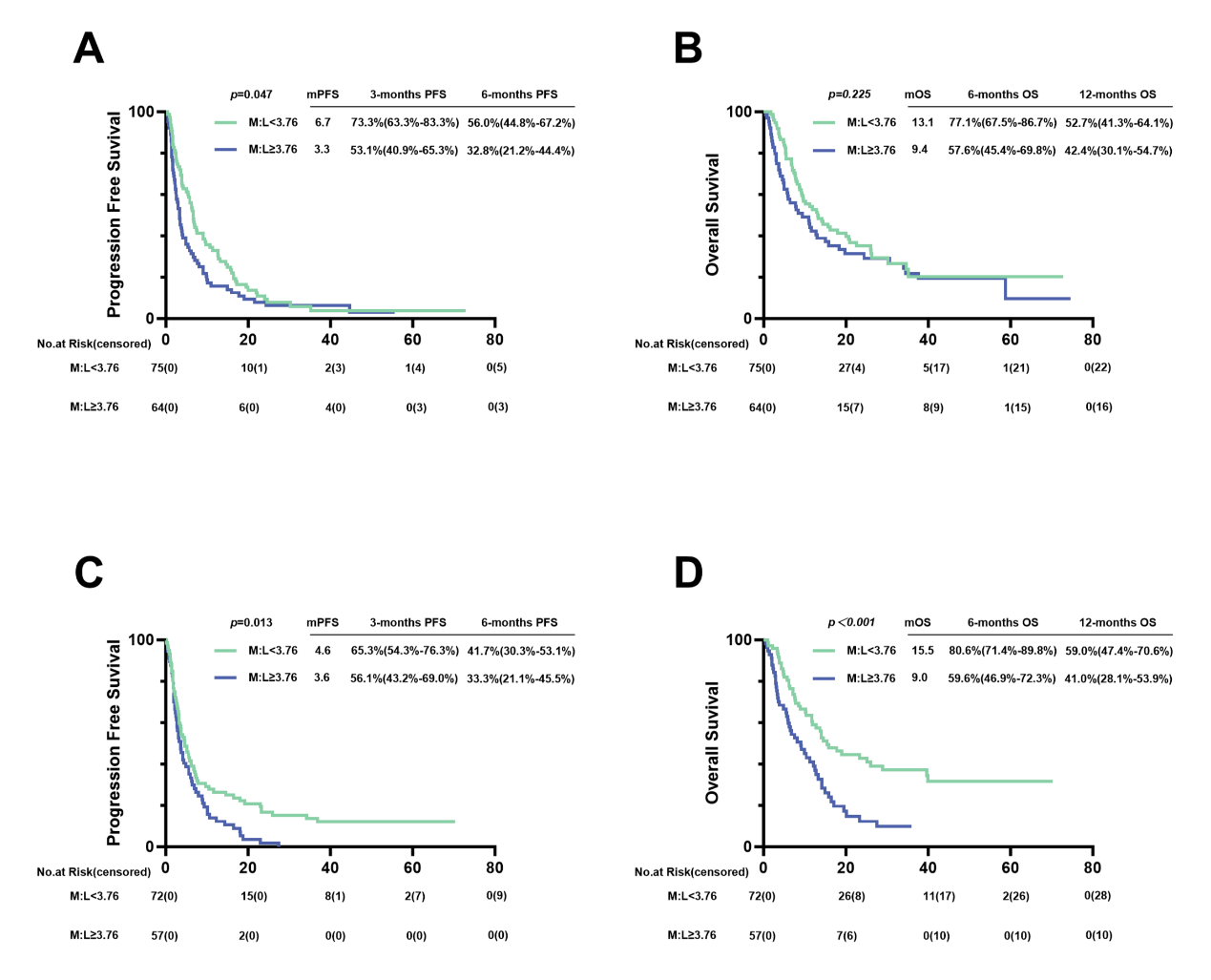


**Supplementary Figure 5.** PFS (A)and (B) OS of AGC patients treated with PD-1 inhibitor aged 59 years or older. PFS (C)and (D) OS of AGC patients treated with PD-1 inhibitor younger than 59 years. Subgroup analysis by age showed that high M:L was statistically significantly associated with poor clinical prognosis. Abbreviations: PFS: progression free survival; OS: overall survival; PD-1: programmed cell death-1.


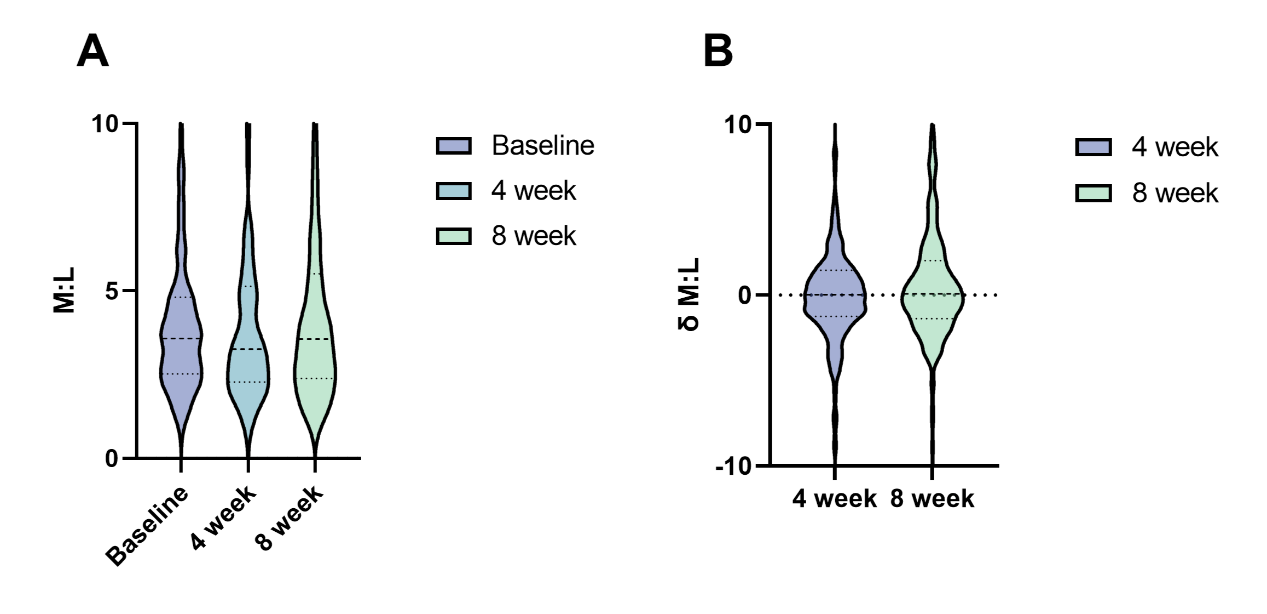


**Supplementary Figure 6.** Dynamic changes of M:L after treatment with PD-1 inhibitors. (A) Distribution of M:L values at baseline, 4 weeks and 8 weeks after receiving PD-1 inhibitors. (B) Distribution of M:L difference from baseline at 4 and 8 weeks after receiving PD-1 inhibitors. Abbreviations: PD-1: programmed cell death-1; δ changes:(4weeks or 8weeks – baseline).

| Supplementary Table 1 Association of δ M:L Changes at 4 Weeks (4 Weeks – Baseline) and progressive disease after 3 months of immunotherapy. | | |
| --- | --- | --- |
| After the first immunotherapy | OR(95% CI) N=225 | P value |
| 3 months | 1.091(1.028-1.157) | 0.004 |

| Supplementary Table 2 Association of δ M:L Changes at 8 Weeks (8 Weeks – Baseline) and death after 6 months of immunotherapy. | | |
| --- | --- | --- |
| After the first immunotherapy | OR(95% CI) N=207 | P value |
| 6 months | 1.089(1.011-1.173) | 0.024 |
